# Long-term survival of high-grade primary peritoneal papillary serous adenocarcinoma: a case report and literature review

**DOI:** 10.1186/s12957-017-1134-3

**Published:** 2017-04-11

**Authors:** Jingping Yuan, Liang He, Bing Han, Yan Li

**Affiliations:** 1 Department of Pathology, Renmin Hospital of Wuhan University, Wuhan, 430060 Hubei People’s Republic of China; 2 Department of Peritoneal Cancer Surgery, Beijing Shijitan Hospital Affiliated to the Capital Medical University, Beijing, 100038 People’s Republic of China; 3 Department of Pathology, Penn State Milton S. Hershey Medical Center, Penn State College of Medicine, Hershey, PA USA

**Keywords:** PPPSC, Lung metastasis, Long-term survival, CRS + HIPEC, Hyperthermic intrapleural chemotherapy

## Abstract

**Background:**

Primary peritoneal papillary serous carcinoma (PPPSC) is an uncommon disease which has a high malignancy and a poor prognosis.

**Case presentation:**

We report here a long-term survival case of PPPSC with postoperative lung metastasis. A 62-year-old female patient with PPPSC was administered two cycles of neoadjuvant chemotherapy (NAC) followed by cytoreductive surgery (CRS) plus hyperthermic intraperitoneal chemotherapy (HIPEC) and six cycles of platinum-based (docetaxel + carboplatin) intraperitoneal chemotherapy postoperatively. The patient reached a complete remission at the completion of primary treatment. Malignant thoracic effusion and lung metastasis developed 5 months after the treatment. The patient underwent video-assisted thoracoscopic surgery plus hyperthermic intrapleural chemotherapy.

**Conclusions:**

Up to present, the patient has been survived with tumor for over 86 months with a good performance status, with only encapsulated effusion found at the latest follow-up. As a relatively new regime, the application of CRS + HIPEC in our patient has been proved example for MPE management, although more large-scale studies are needed to substantiate its efficiency and safety.

## Background

Primary peritoneal papillary serous carcinoma (PPPSC) is an uncommon epithelial tumor which is histologically similar to ovarian papillary serous carcinoma, and the clinicopathological pattern is mainly that of adenocarcinoma [1]. Since the first case reported in 1959, only about 500 cases have been documented. Roffers et al. [2] reported that in the USA, the morbidity was 0.3 per million during 1992 and 1997. The prognosis of high-grade PPPSC is in generally poor, with a median overall survival (OS) ranging from 21 to 23.5 months [3–6]. The fact that very few cases of long-term survival have been reported has testified the poor prognosis to some extent. Intraperitoneal dissemination without ovarian involvements usually present when the diagnosis of PPPSC is made. Other unusual sites of metastasis, such as the main bronchus and brain parenchyma, and remote lymph node involvement have been reported [7–9]. To our knowledge, metastatic PPPSC to the lung has not yet been reported.

## Case presentation

**Fig. 1:**
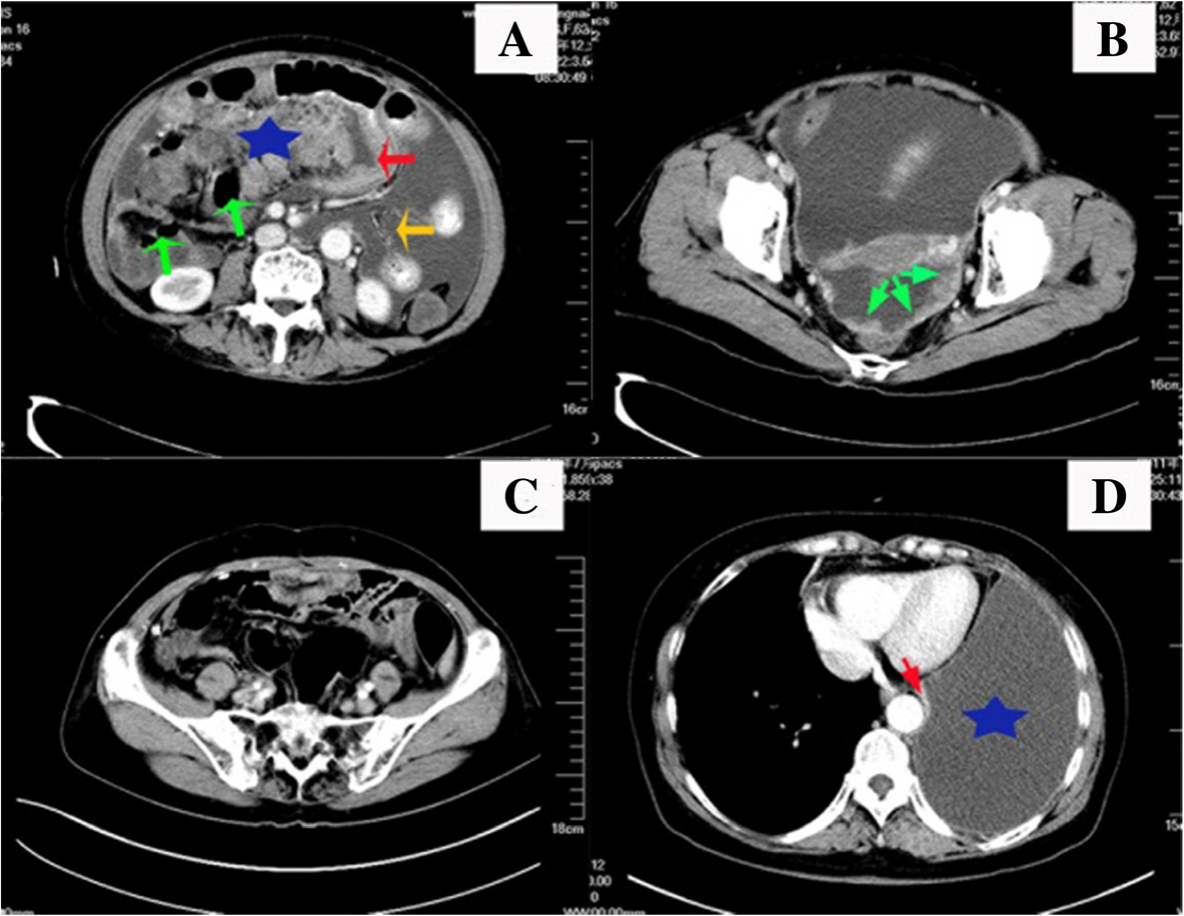
**a** Intravenous and oral contrast-enhanced CT scan shows ascites (*orange arrow*) and omentum cake (*star*) which partially encases intestinal tract (*green arrows*) and adheres to and presses the stomach (*red arrow*). **b** The pelvic peritoneum thickening (*green arrows*), no ovarian and regional lymph nodes involvement was found. **c** No abdominal recurrence when lung metastasis was found. **d** Massive left pleural cavity effusion (*star*) and atelectasis left lower lobe (*red arrow*)

The patient was a 62-year-old female who was referred to our clinic in December 2008 because of a 10-day history of abdominal distention and concomitant nausea and vomiting. She had no family history of ovarian or breast cancer. Physical examinations revealed abundant ascites. Gastrointestinal endoscopy and colonoscopy identified no neoplastic lesion. Abdominopelvic CT scan showed massive ascites, slight bilateral pleural effusions, an omentum lumppartially encasing the intestinal tract, and peritoneum thickening. No evidence of ovarian involvement was found (Fig. 1a, b). Laboratory studies indicated an elevated serum CA-125 level (6222.4 U/ml, normal range 0–35 U/ml). Cytology study of ascites revealed malignant cells. Exploratory laparotomy found a large omentum cake (20 × 10 cm) displacing the stomach, transverse colon, and small bowel. Numerous peritoneal implants scattered subphrenic space, mesostenium, and pelvic floor peritoneum (maximum 3 × 2 cm). The uterus, ovaries, and fallopian tubes appeared macroscopically uninvolved, and no hepatic metastasis was seen. An intraperitoneal infusion pump was embedded subcutaneously, followed by intraperitoneal chemotherapy with docetaxel (80 mg/m^2^) and carboplatin (AUC 6), and intravenous neoadjuvant chemotherapy (NAC) with paclitaxel (80 mg/m^2^) and cisplatin (60 mg/m^2^) on the third postoperative day.

**Fig. 2:**
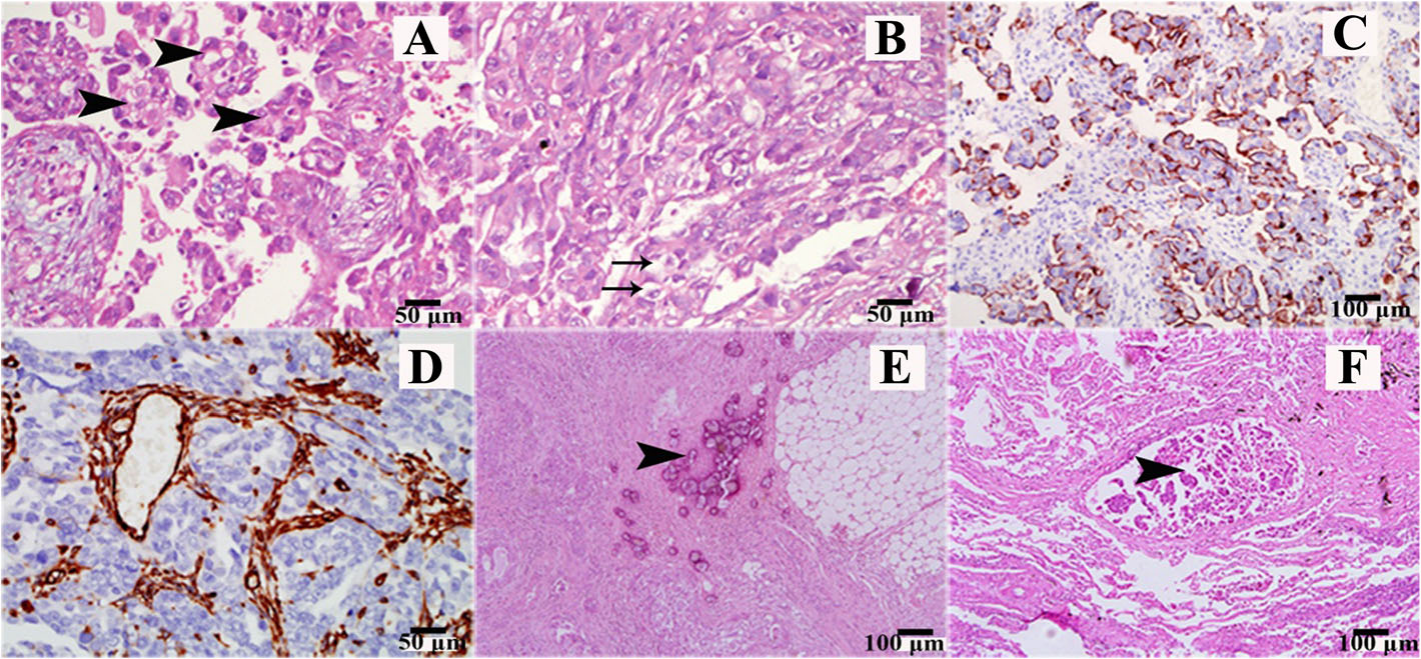
Pathological analysis on the tumor. **a** Papillary growth pattern (*arrows*) of the tumor. **b** Poor differentiated structure. *Arrows* show atypical cells with large nuclei and prominent nucleoli. **c** Result of immunohistochemical staining for CA-125. **d** Result of immunohistochemical staining for vimentin. **e** Calcifications (*also called psammoma bodies*). **f** Metastatic tumor nodule to the left lung

After two cycles of the bi-directional neoadjuvant chemotherapy, the serum CA-125 level was significantly dropped and ascites decreased remarkably. A cytoreductive surgery (CRS) + hyperthermic intraperitoneal chemotherapy (HIPEC) procedure was performed as previously described [10] 40 days later when the general conditions of the patient improved significantly. The peritoneal carcinoma index (PCI) was evaluated as 19. The surgical procedures involved omentectomy, bilateral salpingo-oophorectomy, parietal peritoneotomy, and electrical fulguration of the nodules on the intestinal surface. The completeness of cytoreduction (CCR) was 0, indicating an optimal debulking. Cisplatin 120 mg and mitomycin 30 mg was continuously perfused with 12,000 ml normal saline at 42 °C in 90 min. The patient had uneventful recovery after the operation and the clinicopathological examination of mass specimen denoted a diagnosis of PPPSC (high grade, stage IIIc) (Fig. 2a–e).

**Fig. 3:**
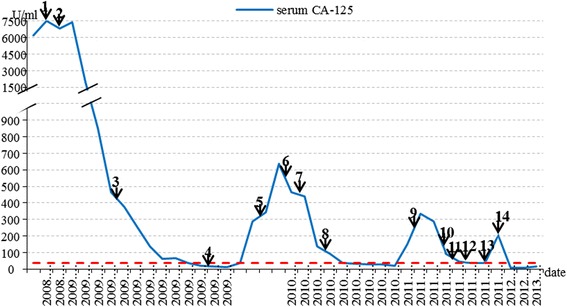
Serum CA-125 level trend. (*1*, *2* show the first and second course of NAC; *3* shows the CRS + HIPEC; *4* shows the completion of primary treatment; *5*, elevated serum CA125, seventh intraperitoneal chemo; *6* shows intrapleural chemo with cisplatin; *7*, intravenous chemo with docetaxel and cisplatin; *8* shows dose-modified intravenous chemo. *9*, *10*, *11*, *12* intrapleural chemo with cisplatin and docetaxel; *13* video-associated thoracoscopic surgery plus hyperthermic intrapleural chemo; *14* shows intrapleural chemo)

Adjuvant intraperitoneal chemotherapy was commenced 22 days after CRS + HIPEC, with docetaxel (60 mg/m^2^) plus carboplatin (AUC 6), every 3 weeks for six cycles. The patient had complete clinical remission (CR) at the completion of the chemotherapy. Pleural effusion completely disappeared. Thereafter, the patient was followed up on a regular basis, which included serum CA-125 levels monitoring at every follow-up visit (Fig. 3 shows the serum CA-125 curve). One year later (Feb. 2010), the serum CA-125 elevated again without sign of relapse. The seventh cycle of aforementioned intraperitoneal chemotherapy was given. Five months later, the patient was hospitalized because of progressing dyspnea. The CT scan showed massive left pleural effusion and atelectasis of the left lower lobe (Fig. 1d). No abdominal recurrence was detected (Fig. 1c) and serum CA-125 level increased further (Fig. 3). Thoracentesis was performed with malignant cells revealed by cytologic examination. The CA-125 level in effusion was >10,000 U/ml. The pleural effusion was not well controlled with intravenous docetaxel-cisplatinum or intrapleural cisplatin, as indicated by progressively increase in CA-125 levels and continuous positive cytologic finding in effusion. Grade II myelosuppression and grade IV gastrointestinal reactions were soon developed, which remained unchanged even with modified dose of chemotherapeutic agents. When the patient recovered from the SAE, a television-assisted thoracoscopy and intrapleural hyperthermic perfusion chemotherapy were performed. Biopsies of the parietal pleura and small nodules on both the visceral pleura and the diaphragm revealed lung metastasis by serous papillary carcinoma (Fig. 2f). Two months after the biopsy, one cycle of intrapleural chemotherapy (oxaliplatin 50 mg) was administered. Thereafter, the patient declined further chemotherapy but adopted traditional Chinese medicine. She has been followed up on a regular basis. By the time of preparation of the manuscript, patient has been survived over 7 years with performance score 1 and an intermediate encapsulated effusion on the left chest, which did not interfere with her daily life.

## Discussion

PPPSC is an uncommon disease. Clinically, the disease presents with gastrointestinal symptoms and general abdominal discomfort. Despite appetite loss, the patients usually undergo weight gaining as the result of ascites [11]. CT scan usually demonstrates ascites, peritoneal nodules, and omental thickening, but seldom identifies original tumors. The majority of patients have an elevated level of serum CA-125 [12], but the preoperative serum CA-125 levels have no significant predictive value for OS [5].

Due to the common embryologic origins, PPPSC and ovarian serous papillary carcinoma share similar gross, histopathologic, and immunohistochemical features. The following criteria to discriminate PPPSC from ovarian papillary serous carcinoma has been suggested by Gynecologic Oncology Group in 1993: (1) both ovaries must be normal in size or enlarged by a benign process; (2) the involvement in extraovarian sites must be greater than the involvement on the surface of either ovary; (3) microscopically, the ovarian component must be nonexistent, confined to ovarian surface epithelium with no evidence of cortical invasion, or involving ovarian surface epithelium and underlying cortical stroma but with tumor size less than 5 × 5 mm; and (4) histological and cytological characteristics of the tumor must be predominantly of the serous type that is similar or identical to ovarian serous adenocarcinoma of any grade [13]. The immunohistochemistry (IHC) expression by PPPSC includes CD15(+), CK7(+), S-100(+), CA125(+), CK20(−), ER(±), PR(±), and CEA(−) [6]. PAX8 and claudin-4 have been being investigated as IHC markers for discriminating peritoneal papillary serous carcinomas and peritoneal epithelioid mesotheliomas [14].

Since PPPSC and ovarian serous cancer had similar response rate and overall survival with similar treatments [15], therefore, the management strategies for stage III/IV ovarian cancer have been recommended by NCCN for primary peritoneal carcinomas. The upfront debulking and following platinum-based adjuvant chemotherapy is considered as a standard treatment. Neoadjuvant chemotherapy (NAC) followed by interval debulking has not been proved to be beneficial to the overall survival, but is applied when the tumor is unresectable, and it can reduce frequency of surgery-related complications [16]. In recent years, CRS + HIPEC have been established for the intervention of peritoneal carcinoma. Taking the combined advantages of the surgery to remove macroscopic tumor and the regional chemotherapy to eliminate microscopic tumor cells, the procedure has been proved to improve OS greatly [10]. Table 1 lists some reports within the latest 10 years.

**Table 1:** Literature reports on PPPSC for the past 10 years

Authors	Cases	Grade			Stage^a^	Debulking surgery		Chemo. regimen	MOS^b^ (months)	PFS (months)
		1	2	3		Optimal	Not			
Bloss [3]	36	3	15	18	III/IV	NA		CP	22	11
Iavazzo [11]	9		NA		III/IV	3	6	Carboplatin or TP	30	NA
Roh [20]	22		NA		IIIc/IV	17	5	Platinum-based	23.1	13.8
Morita [21]	11		NA		NA	5	6	TC or CAP or TP	22	NA
Liu [6]	22	1	10	11	III/IV	18	4	Platinum-based	21	NA
Eisenhauer [22]	43	2	5	36	IIIc/IV	29	14	Platinum	42	17

*NA* not available, *CP* cisplatin/cyclophosphamide, *TP* cisplatin/paclitaxel, *TC* carboplatin/paclitaxel, *CAP* cisplatin/doxorubicin/cyclophosphamide

^a^The majority of cases

^b^Median overall survival

With regard to the prognostic factors of PPPSC, Eltabbakh et al. [5] suggested that age <70 at diagnosis, performance status ≤1, and residual tumor size ≤1 cm had significant impact on OS. Schmeler et al. [17] reported that although low-grade serous primary peritoneal carcinomas have longer OS, they had higher drug resistance rate to conventional chemotherapy. In recent years, several literatures reported that the regression coefficient of CA-125 during the preoperative neoadjuvant chemotherapy was predictive of overall survival, time to the second-line treatment, time to CA-125 progression, and of the optimal cytoreduction rate at interval debulking surgery [18]. On the ground of all these findings, the fast preoperative serum CA-125 regression rate, optimal debulking, good performance status, and age at diagnosis may all have contributed to our patient’s long-term survival.

CA-125 monitoring is generally recommended if the levels are initially elevated. CT scan and other radiological imaging should also be performed if necessary. On the ground of the NCCN guideline for ovarian cancer, it remains controversial whether the patients who are in complete remission but present with merely an increasing CA-125 with negative symptom or negative radiological findings (called biochemical relapse) need to be treated. We gave additional cycle of intraperitoneal chemotherapy to our patient after she had reached the stage of biochemically relapsing. The serum CA-125 continued rising (Fig. 3), which followed by pleural effusion. Though the intravenous docetaxel plus cisplatin effectively reduced the serum CA-125 level, the pleural effusion was not well controlled and cytologic analysis of the pleural fluid was persistently positive for malignant cells. Malignant pleural effusion (MPE) has been considered to be associated with a poor prognosis. CRS + HIPEC regime has been proved to be of survival benefit for this group of patients [19].

For our patient, neoadjuvant chemotherapy was followed by a dramatic CA-125 level drop and less postoperative complications, which testified the effect of NAC on reducing tumor mass and decreasing operation-related complications. However, whether NAC provides survival benefit needs to be further studied. When the serum CA-125 levels were analyzed retrospectively, we found the CA-125 levels before the next course of chemotherapy were lower than that of the previous hospitalization. If a persistent drop occurs between the two courses of chemotherapies, we could prolong the time interval between the chemotherapies so that the toxic effects of chemotherapy agents can be reduced. According to Isik et al. study [19], 1 year survival of patient with MPE was less than 0.8%. With CRS + HIPEC, our patient has been alive for over 7 years with a good quality of life.

## Conclusions

As a relatively new regime, the application of CRS + HIPEC in our patient has been proved example for MPE management, although more large-scale studies are needed to substantiate its efficiency and safety.

## Abbreviations

CCR: Completeness of cytoreduction
CRS: Cytoreductive surgery
HIPEC: Hyperthermic intraperitoneal chemotherapy
IHC: Immunohistochemistry
NAC: Neoadjuvant chemotherapy
OS: Overall survival
PCI: Peritoneal carcinoma index
PPPSC: Primary peritoneal papillary serous carcinoma
